# 5-Aminolevulinic Acid-Based Radiodynamic Therapy for Malignant Gliomas: A Conceptual Framework for Mitochondria-Centered Mechanisms, Target Cell States and Translational Perspectives

**DOI:** 10.3390/life16020318

**Published:** 2026-02-12

**Authors:** Junkoh Yamamoto

**Affiliations:** Department of Neurosurgery, University of Occupational and Environmental Health, Iseigaoka, Yahatanishi-ku, Kitakyushu 807-8555, Fukuoka, Japan; yama9218@med.uoeh-u.ac.jp; Tel.: +81-93-691-7257; Fax: +81-93-691-8799

**Keywords:** 5-aminolevulinic acid, protoporphyrin IX, radiodynamic therapy, radiotherapy, mitochondria, reactive oxygen species, binary approach, glioblastoma

## Abstract

5-Aminolevulinic acid (5-ALA) is a naturally occurring heme precursor with a favorable safety profile and is widely used for fluorescence-guided resection of malignant gliomas. Exogenous administration of 5-ALA results in the selective intracellular accumulation of protoporphyrin IX (PpIX), predominantly within tumor cell mitochondria, reflecting tumor-specific alterations in cellular metabolism and heme biosynthetic pathways. Historically, the radiosensitizing potential of 5-ALA was considered limited, as 5-ALA itself is not a porphyrin and intracellular PpIX levels are lower than those achieved with classical porphyrin-based agents, such as hematoporphyrin derivatives or porfimer sodium. Recent experimental and translational studies have challenged this view by demonstrating that the interactions between 5-ALA-induced PpIX and ionizing irradiation elicit biologically significant antitumor effects. This emerging concept has been termed radiodynamic therapy (RDT) and represents a therapeutic paradigm distinct from conventional DNA-centered radiosensitization. Accumulating evidence suggests that 5-ALA-based RDT induces mitochondria-centered oxidative stress through both immediate and delayed reactive oxygen species generation, thereby linking metabolic vulnerability to the radiation response. In this review, we summarize the current mechanistic insights into 5-ALA-based RDT, particularly mitochondrial dysfunction and oxidative stress amplification. We also discuss the translational implications and future perspectives for integrating 5-ALA-based RDT into multimodal treatment strategies for malignant gliomas.

## 1. Introduction

Malignant gliomas are highly aggressive and locally invasive brain tumors associated with an extremely poor prognosis. Despite multimodal therapy, including maximal extent of resection (EOR) and concomitant radiochemotherapy, the median survival of patients with glioblastoma (GBM) remains only 12–14 months [1,2]. Owing to their highly infiltrative nature, most GBMs disseminate into the surrounding normal brain tissue at the time of diagnosis, resulting in a high recurrence rate [3]. Up to 85% of GBM recurrences arise within a prior radiation field [4], underscoring the intrinsic radioresistance of infiltrative glioma cells and the clinical need for strategies that effectively target residual and invasive disease. This recurrence pattern highlights a fundamental limitation of conventional DNA-centered radiotherapy and emphasizes the need for alternative or complementary strategies capable of targeting infiltrative tumor cell populations beyond image-defined lesions. 5-Aminolevulinic acid (5-ALA) induces the intracellular accumulation of protoporphyrin IX (PpIX), a photosensitizing porphyrin that localizes predominantly to tumor cell mitochondria and has been widely utilized in oncology [5,6,7,8,9,10,11,12]. 5-ALA fluorescence-guided surgery (FGS) has improved intraoperative visualization and enabled a greater EOR than conventional microsurgical approaches in malignant glioma surgery [13], and has consequently been adopted worldwide.

Since the 1950s, porphyrin compounds such as hematoporphyrin derivatives (HpD) and porfimer sodium have been recognized as photosensitizers and radiosensitizers, prompting early clinical investigations of porphyrins as adjuvants to radiotherapy [14,15,16,17]. In contrast, relatively few in vitro studies have examined the interaction between ionizing irradiation and 5-ALA [18,19]. Because 5-ALA is not a porphyrin and the intracellular levels of 5-ALA-induced PpIX are lower than those achieved with HpD or porfimer sodium, the radiosensitizing capacity of 5-ALA has long been considered negligible [20]. Consequently, porphyrin-based research has shifted toward the development of photosensitizers rather than radiosensitizers. Consequently, the potential biological significance of interactions between 5-ALA-induced PpIX and ionizing irradiation has historically been underestimated.

Challenging this prevailing view, our previous studies demonstrated that although the intrinsic radiosensitizing effect of 5-ALA is modest, fractionated ionizing irradiation markedly amplifies this effect in vitro [21]. Moreover, repeated irradiation combined with 5-ALA significantly enhances host antitumor immunity and suppresses tumor growth in in vivo experimental glioma models [22]. Subsequent experimental studies reported similar synergistic interactions between ionizing irradiation and 5-ALA across a range of malignancies, including melanoma, breast cancer, prostate cancer, colorectal cancer, lung cancer, central nervous system lymphoma, and meningioma [23,24,25,26,27,28,29]. Recently, this phenomenon has increasingly been conceptualized as radiodynamic therapy (RDT), a therapeutic paradigm that extends beyond classical radiosensitization [30,31,32]. Importantly, RDT represents a conceptual shift from purely DNA-centered radiation effects to broader cellular vulnerabilities, including oxidative stress, metabolic perturbations, and mitochondrial dysfunction.

Because studies on 5-ALA-RDT are limited, and causal evidence directly linking mitochondrial oxidative stress to tumor cell death in radiation biology has not yet been fully established, this review does not aim to present definitive mechanistic conclusions. Instead, we sought to critically synthesize the existing experimental, translational, and early clinical data to propose a coherent mechanistic framework for 5-ALA-RDT. Understanding 5-ALA-RDT requires integrating two traditionally distinct perspectives: photochemical principles from photodynamic therapy (PDT) and emerging concepts in radiation biology that go beyond nuclear DNA damage. Accordingly, this review explicitly distinguishes between mechanisms that have been experimentally demonstrated and hypothesis-driven interpretations that incorporate the currently available evidence. These interpretations are presented not as definitive causal mechanisms but as a structured conceptual framework intended to guide future experimental validation, critical discussion, and rational clinical development of 5-ALA-RDT.

## 2. Interaction Between 5-ALA-Induced PpIX and Ionizing Irradiation

RDT integrates ionizing radiation with radiosensitizers, predominantly porphyrinoid compounds, to induce oxidative stress and subsequent tumor cell death. Therefore, RDT is conceptually analogous to PDT, which combines light irradiation with photosensitizers. However, although ionizing radiation and visible light are both electromagnetic waves, they differ substantially in photon energy, tissue penetration, and the spatiotemporal characteristics of their biological effects. Previous studies have demonstrated that PpIX enhances the generation of reactive oxygen species (ROS), including hydroxyl radicals, superoxide anions, and singlet oxygen, via radiation-induced water radiolysis during irradiation [33]. In contrast, singlet oxygen in PDT is predominantly produced via a type II photochemical reaction following the light excitation of the photosensitizer [34,35]. Additionally, other research has demonstrated that ionizing irradiation induces degradation of PpIX (“radiobleaching”), which is conceptually analogous to photobleaching observed during PDT [36]. Collectively, these observations support the notion that PpIX interacts directly with ionizing radiation and functions as a radiosensitizer during RDT.

Regarding ROS-related dynamics during treatment, we previously demonstrated real-time in vivo monitoring of singlet oxygen production in experimental gliomas during 5-ALA-PDT by detecting near-infrared luminescence derived from singlet oxygen using a photomultiplier-based system [37]. Building on the oxygen-dependent delayed fluorescence properties of light-excited PpIX and the preferential mitochondrial accumulation of 5-ALA-induced PpIX, the PpIX triplet-state lifetime technique (PpIX-TLST) was developed to enable the quantitative assessment of mitochondrial oxygen tension [38]. Recent studies have quantified intracellular oxygen transients in vivo during ultrahigh-dose-rate FLASH radiotherapy using 5-ALA-induced PpIX as an intracellular oxygen sensor based on PpIX-TLST [39]. Together, these observations provide experimental support for the feasibility of investigating mitochondrial redox and oxygen dynamics in vivo in the presence of PpIX during irradiation, and are consistent with a potential mechanistic role for mitochondrial ROS generation in 5-ALA-RDT.

At the system level, transcriptomic profiling in a mouse subcutaneous melanoma model demonstrated that 5-ALA-RDT induced greater downregulation of cell cycle-related pathways and upregulation of oxidative stress- and antitumor immunity-associated genes than irradiation alone [40]. Compared to irradiation alone, we consistently observed that 5-ALA-RDT suppressed tumor growth and promoted the accumulation of Iba-1-positive macrophages with phagocytic features at the boundary between coagulative necrosis and viable tumor tissue in a rat subcutaneous glioma model [22]. Notably, repeated administration of 5-ALA alone suppressed tumor growth and induced Iba-1-positive macrophage aggregation at the tumor surface [22]. Furthermore, the results of our in vitro experiments demonstrated that 5-ALA can disrupt immune tolerance in glioma cells by suppressing prostaglandin E_2_ production [41]. In addition, a recent study demonstrated that 5-ALA-RDT with single-dose X-ray irradiation induced significant tumor regression in a murine subcutaneous colon cancer model [42]. Histopathological analyses revealed disruption of tumor cell islands accompanied by increased infiltration and a close spatial association of Iba-1-positive macrophages with tumor cells in the 5-ALA-RDT group. Transcriptomic profiling using microarray analysis further identified the coordinated downregulation of gene sets associated with DNA repair, cell cycle regulation, autophagy, metabolic pathways, and immune evasion compared to the control and irradiation-alone groups [42]. These findings suggested that 5-ALA-RDT elicits pleiotropic biological responses that extend beyond its direct cytotoxic effects. Although direct investigations of the immunological mechanisms of 5-ALA-RDT remain extremely limited, recent findings from the PDT field provide important conceptual context for potential immunomodulatory effects. A recent study using a bilateral subcutaneous colon cancer mouse model demonstrated that 5-ALA-based PDT can induce immunogenic cell death (ICD) and promote systemic antitumor immune responses [43]. Several key observations are made in this study. First, 5-ALA-PDT-induced downregulation of AKT signaling promotes the release of damage-associated molecular patterns (DAMPs), including calreticulin, leading to the activation of dendritic cells. Second, 5-ALA-PDT simultaneously induces autophagy, which partially suppresses ICD signaling. Third, the pharmacological inhibition of autophagy using chloroquine enhanced ICD induction following 5-ALA-PDT. As a consequence, combined treatment with 5-ALA-PDT and chloroquine enhanced the maturation of dendritic cells and cytotoxic T lymphocytes, leading to significant tumor growth suppression at the primary tumor treatment site. Importantly, this immune activation was marked by an accumulation of mature dendritic cells and cytotoxic T lymphocytes in the spleen, an increase in systemic cytokine release (including TNF-α and IFN-γ), and inhibition of tumor growth at the contralateral, non-treated tumor site [43].

Although these findings were obtained in the context of PDT rather than RDT, they support the broader concept that porphyrin-mediated oxidative stress can induce ICD and promote systemic antitumor immunity. However, due to fundamental differences in radiation energy, dominant ROS, oxidative capacity, and the spatial distribution of oxidative stress, the biological consequences of 5-ALA-RDT are expected to differ substantially from those of 5-ALA-PDT. While similar immune-modulating mechanisms may plausibly contribute to the therapeutic effects of 5-ALA-RDT, direct experimental validation of these mechanisms in a radiodynamic setting is currently lacking.

## 3. Oxidative Stress and Mitochondrial Damage in 5-ALA-RDT

Before discussing delayed oxidative stress or radiodynamic amplification, it is essential to summarize the experimentally established evidence demonstrating that ionizing irradiation directly perturbs mitochondrial structure and cell-cycle regulation through mechanisms that are, at least in part, independent of canonical apoptosis. In general, ionizing irradiation induces tumor cell death through two principal pathways: (i) direct interactions with nuclear DNA (“direct effects”), and (ii) indirect effects mediated by water radiolysis and subsequent ROS generation—, particularly hydroxyl radicals—, that damage biomolecules, including nuclear DNA (“indirect effects”), ultimately leading to reproductive cell death [44,45]. These primary physicochemical reactions occur within an extremely short period of irradiation [46]. However, our previous in vitro analyses demonstrated that immediately after 5-ALA-RDT, ROS signals were predominantly localized to the cytosol rather than the nucleus and spatially colocalized with 5-ALA-PpIX in glioma cells [21]. Given that mitochondria occupy a substantial fraction of the cellular volume (approximately 4–25%, depending on the cell type) [47] and that 5-ALA-induced PpIX preferentially accumulates within the mitochondria, mechanistic analyses of 5-ALA-RDT should not be confined to nuclear DNA damage alone. Rather, oxidative stress within the cytosol–mitochondrial compartment warrants focused consideration as a central determinant of biological responses in 5-ALA-RDT, as this compartment likely represents a major site of radiation-induced redox perturbations.

The mitochondrial matrix is characterized by a relatively elevated redox environment compared to that in the cytosol, reflecting constitutive ROS production associated with oxidative phosphorylation [48,49]. A substantial fraction of intracellular ROS— including those generated following low-dose ionizing irradiation—is thought to originate from the mitochondria as a consequence of electron leakage from the mitochondrial electron transport chain [50]. Excessive or dysregulated mitochondrial ROS production can result in mitochondrial dysfunction and initiate mitochondria-mediated intracellular and extracellular signaling cascades, including those involved in immune and inflammatory responses [51,52]. Although mitochondrial DNA (mtDNA) represents only ~0.25% of the total cellular DNA, nearly all mtDNA (except the D-loop) encodes proteins essential for mitochondrial function [53,54]. Moreover, mtDNA is particularly vulnerable to oxidative damage because of limited histone protection and relatively inefficient repair mechanisms [55,56]. Historically, radiation biology has focused primarily on nuclear targets; however, accumulating evidence indicates that ionizing radiation can also modulate mitochondrial membrane potential, respiration, adenosine triphosphate (ATP) production, and mitochondrial ROS generation in tumor cells [57,58]. With respect to radiation-induced alterations in mitochondrial dynamics, a seminal series of studies has demonstrated that ionizing irradiation triggers the translocation of the mitochondrial fission protein dynamin-related protein 1 (Drp1) from the cytosol to the mitochondria, thereby promoting mitochondrial fission and subsequent mitochondrial fragmentation in a time-dependent manner [59]. Excessive mitochondrial fragmentation ultimately leads to mitochondrial catastrophe (MC), a form of mitochondrial dysfunction characterized by profound structural disorganization and the loss of functional integrity. Importantly, radiation-induced MC does not primarily activate the intrinsic apoptotic pathway initiated by the cytosolic release of mitochondrial proteins or the immediate collapse of mitochondrial bioenergetics. Instead, this process proceeds independently of canonical mitochondrial apoptotic signaling. Mechanistic analyses revealed that Drp1-dependent mitochondrial fission interferes with the centrosome duplication cycle and mitotic progression, implicating key cell-cycle regulators, including polo-like kinase 1 (Plk1) and cyclin-dependent kinase 2 (CDK2). Consistent with this model, pharmacological or genetic suppression of Drp1 preserved cyclin B1 levels after irradiation and significantly attenuated mitotic catastrophe, further supporting the functional link between mitochondrial dynamics and radiation-induced cell-cycle dysregulation. Notably, Drp1 activation peaks at approximately 8–12 h after ionizing irradiation, whereas overt mitochondrial fragmentation becomes evident later, typically between 12 and 24 h post-irradiation. This temporal dissociation indicates that radiation-induced mitochondrial responses are not instantaneous events but rather evolve progressively and persist over time following the initial radiation insult [59]. Collectively, these findings demonstrate that ionizing radiation induces a sustained and temporally progressive mitochondrial response that extends well beyond the initial physical interactions between radiation and cellular targets. These time-dependent mitochondrial perturbations provide a conceptual foundation for understanding the delayed biological consequences of irradiation, which may critically influence downstream cell fate decisions.

## 4. Fractionation-Dependent Radiodynamic Effects and Temporal Mitochondrial ROS Kinetics

Despite increasing evidence supporting the radiodynamic effects of 5-ALA in tumor cells [24,25,28,29,32,60,61], the optimal irradiation parameters for 5-ALA-RDT, including radiation dose, fractionation schedule, and photon energy, have not yet been established. Moreover, although mitochondrial ROS generation and subsequent mitochondrial dysfunction have been strongly implicated in the biological effects of 5-ALA-RDT, the precise molecular mechanisms linking mitochondrial ROS production to tumor-specific lethality following ionizing irradiation remain incompletely defined. In addition, the temporal kinetics of ROS production—, including magnitude, duration, and recovery dynamics—, remain largely unresolved in the context of 5-ALA-RDT.

Given these limitations, this section adopts a hypothesis-driven interpretative approach that integrates currently available experimental and translational evidence. Rather than asserting definitive causal mechanisms, we propose a coherent conceptual framework intended to guide the rational optimization of irradiation timing and fractionation strategies in 5-ALA-RDT. Preclinical studies employing fractionated irradiation provide important, albeit heterogeneous, insights into potential radiodynamic effects. We previously demonstrated that, in a rat subcutaneous glioma model, fractionated irradiation at 2 Gy/day for five consecutive days (total dose, 10 Gy) combined with 5-ALA significantly inhibited tumor growth and induced marked accumulation of Iba-1-positive macrophages within the tumor tissue [22]. Similarly, another study in a mouse subcutaneous melanoma model demonstrated that fractionated 5-ALA-RDT (2 Gy/day for five consecutive days, repeated over two courses; total 20 Gy) suppressed tumor growth and induced transcriptomic changes consistent with oxidative stress and immune activation [60]. In addition, a mouse subcutaneous prostate cancer model demonstrated significant antitumor effects with 5-ALA-RDT administered at 4 Gy/day for three consecutive days (total 12 Gy) [25]. In contrast, in an orthotopic brain tumor model inoculated with patient-derived GBM stem cells, 5-ALA-RDT delivered at 2 Gy per fraction five times per week (described as three times weekly on Monday, Wednesday, and Friday; total 10 Gy) did not yield a consistent radiodynamic effect, with substantial interindividual variability in bioluminescence-based tumor burden [62]. This discrepancy highlights the complexity of translating fractionation paradigms across tumor models and suggests that the biological context, including mitochondrial stress responses and ROS kinetics, may critically influence therapeutic outcomes. In addition to fractionated irradiation, several studies have demonstrated measurable radiodynamic effects under single-dose irradiation. A murine subcutaneous colon cancer model showed marked tumor growth suppression following a single 12 Gy X-ray irradiation combined with 5-ALA, accompanied by enhanced antitumor immune responses and transcriptomic signatures of immune activation [42]. Similarly, another in vivo study demonstrated significant tumor growth inhibition in a mouse lung cancer model treated with a single 4 Gy fraction of 15-MV photon irradiation in combination with 5-ALA [29]. Collectively, these findings indicate that both single-dose and fractionated irradiation regimens can elicit radiodynamic effects when combined with 5-ALA; however, the determinants of their efficacy remain poorly understood.

We further confirmed that intracellular levels of 5-ALA-induced PpIX in viable glioma cells remain stable or may even increase following radiotherapy [63]. From a clinical perspective, these observations suggest that repeated administration of 5-ALA in combination with fractionated irradiation may sustain or augment intracellular PpIX availability within residual tumor cells, thereby potentially reinforcing radiodynamic efficacy. However, this hypothesis requires further experimental and clinical validation.

A fundamental concept underlying redox-based cancer therapy is that ROS exert biphasic and context-dependent effects on tumor cells [64]. Moderate elevations in intracellular ROS levels can promote tumor progression by supporting proliferation, metabolic adaptation, and survival signaling, whereas excessive ROS accumulation beyond a critical threshold induces irreversible cellular damage and cell death [64]. This “ROS threshold” model implies the existence of a tumor-specific lethal oxidative boundary, above which cancer cells can no longer maintain redox homeostasis.

As discussed in previous sections, mitochondrial dysfunction induced by ionizing irradiation is not instantaneous but develops progressively over time, providing a biological basis for delayed oxidative stress. In this context, mitochondrial ROS are often secondary products arising from sustained redox cascades initiated by primary radiation-induced species, including hydroxyl radicals [58,65,66,67]. Specifically, irradiation can induce mitochondrial oxidative stress, leading to mtDNA injury and metabolic perturbations that generate additional ROS—predominantly superoxide—via the dysregulations of mitochondrial bioenergetics. These secondary ROS can damage neighboring mitochondria, propagating oxidative stress throughout the mitochondrial network in a process referred to as “intermitochondrial communication” [68]. Such secondary metabolic events may occur hours after irradiation and contribute to prolonged and delayed ROS production [57,58], which plays a critical role in radiation-induced signaling and cell death pathways [58,69,70].

We have consistently demonstrated that 5-ALA-RDT induces pronounced mitochondrial stress and significantly enhances delayed ROS production in glioma cells in vitro [63,71]. Notably, the administration of 5-ALA after irradiation did not augment delayed ROS generation [63], indicating that substantial mitochondrial accumulation of 5-ALA-induced PpIX at the time of irradiation is a prerequisite for sustained oxidative amplification. These observations suggest that delayed ROS production in 5-ALA-RDT reflects time-dependent mitochondrial dysfunction rather than the persistence of primary radiation-induced radicals. As mitochondrial ROS accumulate beyond the physiological buffering capacity, oxidative injury propagates across the mitochondrial network, ultimately leading to mitochondrial catastrophe characterized by excessive mitochondrial fragmentation and functional collapse. Importantly, the mitochondrial catastrophe induced under these conditions has been shown to interfere with the centrosome amplification cycle and mitotic progression, thereby disrupting cell-cycle control independent of classical apoptotic pathways. In this setting, nuclear DNA damage appears necessary but is insufficient on its own; effective propagation of mitochondrial oxidative stress, convergence toward mitochondrial catastrophe, and subsequent cell-cycle arrest collectively contribute to irreversible tumor cell death in 5-ALA-RDT.

**Figure 1:**
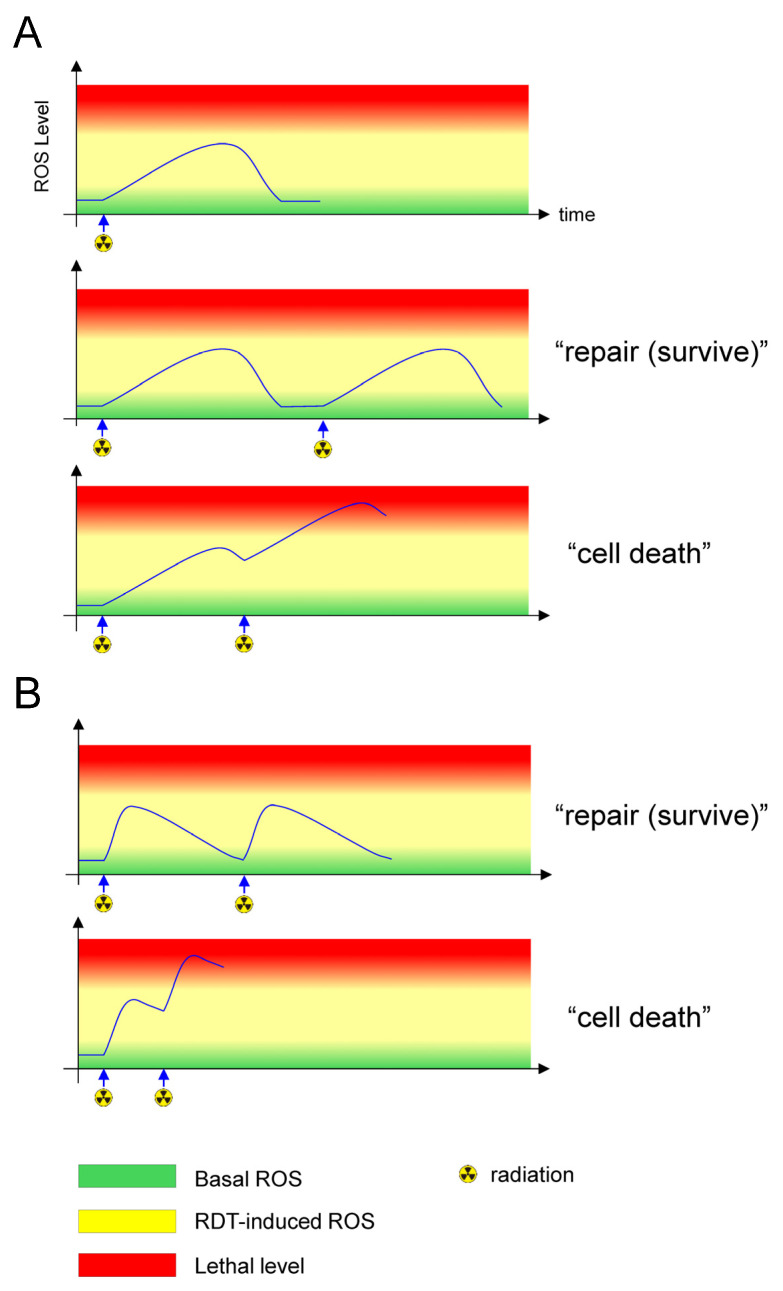
Temporal relationship between delayed ROS production and fractionated ionizing irradiation in 5-ALA-based radiodynamic therapy. Conceptual framework linking delayed reactive oxygen species (ROS) production and timing of fractionated ionizing irradiation in 5-ALA-based radiodynamic therapy (5-ALA-RDT). Unlike classical radiotherapy, in which dose fractionation is primarily intended to spare normal brain tissue, 5-ALA-RDT fractionation is designed to cumulatively elevate mitochondrial oxidative stress to a lethal threshold. (**A**) Glioma and lymphoma. In glioma and lymphoma cells, delayed ROS production increases gradually over time following initial irradiation (upper panel). If subsequent irradiation is delivered only after recovery from mitochondrial oxidative stress, ROS levels fail to reach the lethal threshold, allowing cellular repair and survival (middle panel). In contrast, when subsequent fractions are administered within a shorter interval before delayed ROS resolution, oxidative stress accumulates beyond a lethal level through intermitochondrial communication, ultimately leading to tumor cell death (lower panel). (**B**) Prostate cancer. In prostate cancer cells, delayed ROS production peaks earlier after irradiation. Consequently, applying the same irradiation interval used for gliomas and lymphomas fails to drive ROS production to lethal levels (upper panel). However, shortening the interval between irradiation fractions allows sufficient delayed ROS accumulation, surpassing the lethal threshold and inducing tumor cell death (lower panel). Collectively, this schematic highlights that the optimal timing of fractionated irradiation in 5-ALA-RDT should be tailored to the tumor-specific kinetics of delayed mitochondrial ROS production, rather than applying a uniform fractionation schedule across tumor types.

To achieve irreversible tumor cell death in 5-ALA-RDT, oxidative stress must not only be initiated but also effectively propagated and amplified to exceed a lethal threshold within tumor cells. This requirement highlights the importance of understanding the temporal dynamics of ROS generation—namely, ROS kinetics—rather than considering ROS production as a static or instantaneous event. Despite its potential mechanistic and therapeutic relevance, systematic investigations of ROS kinetics, particularly delayed mitochondrial ROS production, remain extremely limited in the context of 5-ALA-RDT. Consequently, how ROS accumulation evolves over time, how it differs among tumor types, and how it can be modulated to reach tumor-specific lethal levels remain largely unresolved. Regarding ROS kinetics, we observed progressively increasing intracellular ROS levels, reaching a peak within approximately 12 h after 5-ALA-RDT in glioma and lymphoma cell lines [26,63]. Conversely, a study using prostate cancer cells reported an immediate increase in ROS levels after 5-ALA-RDT, followed by a gradual decline within 12 h [25]. These findings suggest that the temporal patterns of ROS generation depend on tumor type and may critically influence therapeutic efficacy. From a mitochondrial perspective, these findings imply that the timing of the subsequent irradiation fractions may be a decisive determinant of the radiodynamic outcome. Specifically, the delivery of additional irradiation before the full recovery of mitochondrial function may promote the effective intermitochondrial propagation of oxidative stress, thereby amplifying mitochondrial ROS accumulation beyond a tumor-specific lethal threshold [26,64] (Figure 1). This concept differs fundamentally from the classical rationale for fractionated radiotherapy, which is primarily designed to spare normal tissues based on DNA repair and repopulation kinetics. In 5-ALA-RDT, fractionation may instead function to temporally integrate mitochondrial oxidative stress, driving irreversible cytotoxicity through sustained mitochondrial dysfunction rather than cumulative nuclear DNA damage alone. Conventionally, fractionated radiotherapy has been explained by the classical “four Rs” of radiobiology (reassortment, repair, reoxygenation, and repopulation), which describe the biological basis of fractionation for tumor control while sparing normal tissues [72]. Subsequently, intrinsic radiosensitivity and, more recently, the reactivation of antitumor immune responses have been proposed as the fifth and sixth Rs, respectively, to account for intertumoral variability in radiation responses [72,73]. More recently, a revised framework distinguishing “DNA damage” from “cellular damage” has emerged, recognizing that irradiation injures not only nuclear DNA but also the plasma membrane and intracellular organelles [74]. Mitochondrial injury can drive mitochondrial reprogramming, including mtDNA damage, ROS leakage, metabolic alterations, and apoptosis-related signaling [74]. Moreover, accumulating evidence suggests that ionizing irradiation can induce mitochondrial DNA damage, which in turn activates cell-intrinsic immune surveillance programs, including the cytosolic accumulation of mitochondrial RNA and activation of the retinoic inducible gene I-mitochondrial antiviral signaling protein (RIG-I-MAVS) axis. These responses may cooperate with nuclear DNA damage signaling to enhance interferon-stimulated gene expression [75]. Thus, ionizing irradiation uniquely targets both nuclear and mitochondrial genomes, positioning mitochondrial DNA damage as a critical amplifier of radiation-induced immune surveillance beyond classical nuclear DNA-centered paradigms.

Taken together, ionizing irradiation in 5-ALA-RDT inevitably induces nuclear DNA damage, while concomitantly imposing substantial mitochondrial injury, which critically contributes to tumor cell death. Fractionated irradiation may therefore act not merely to accumulate DNA lesions, but also to progressively amplify mitochondrial oxidative perturbations beyond a critical threshold, driving mitochondrial reprogramming and irreversible loss of cellular viability. It must be emphasized that direct experimental evidence supporting this mitochondria-centered interpretation in 5-ALA-RDT remains limited. Nevertheless, strict adherence to a DNA-centered radiobiological framework is insufficient to explain the delayed oxidative stress and heterogeneous treatment responses observed in 5-ALA-RDT. Accordingly, integrating mitochondrial biology into radiobiological thinking, rather than replacing established DNA damage paradigms, appears necessary to advance the mechanistic understanding and rational clinical development of 5-ALA-RDT.

## 5. Binary Approach for Functional Mitochondrial Targeting Using 5-ALA and Ionizing Irradiation

Based on the experimental observations summarized above, we proposed a binary conceptual framework for the application of 5-ALA-RDT in cancer treatment. Specifically, although neither 5-ALA nor ionizing irradiation alone can be regarded as a mitochondria-targeted therapeutic modality, their combination in 5-ALA-RDT may functionally converge on the mitochondria, thereby representing a candidate strategy for mitochondria-focused cancer therapy. Mitochondria are the central hubs of cellular metabolism that regulate ATP production, apoptotic signaling, and intracellular ROS generation. Accumulating evidence further indicates that mitochondria actively participate in tumor-immune interactions; for example, mitochondrial transfer within the tumor microenvironment has been implicated in immune evasion [76]. Accordingly, mitochondrial metabolism and signaling pathways have emerged as attractive therapeutic targets in oncology [77,78]. We previously demonstrated the radiodynamic effects of 5-ALA in multiple lymphoma cell lines, including Raji, HKBML, and TK; however, the magnitude of these effects varied substantially across cell types [26]. Notably, Raji cells exhibited a particularly pronounced radiodynamic response, leading us to hypothesize that sustained oxidative stress exceeding a tumor-specific lethal threshold after irradiation is a key determinant of therapeutic efficacy in 5-ALA-RDT, consistent with prior observations in redox-based cancer therapy [79].

Mechanistically, enhanced delayed ROS production mediated by intermitochondrial communication may amplify radiodynamic effects, as exemplified by the response observed in Raji cells. Based on this hypothesis, we postulated that mitochondrial spatial proximity and density may critically influence the efficiency of intermitochondrial communication. In support of this notion, we observed that Raji cells possess significantly higher mitochondrial density compared with other lymphoma cell lines [26]. Furthermore, independent experimental evidence has shown that mitochondrial transplantation into glioma cells increases mitochondrial density and markedly enhances irradiation-induced ROS production—predominantly hydroxyl radicals—under ionizing irradiation [80]. Collectively, these findings suggest that mitochondrial abundance and spatial organization may modulate the amplification of radiation-induced oxidative stress.

Importantly, neither mitochondrial accumulation of PpIX induced by 5-ALA nor ionizing irradiation alone was sufficient to induce mitochondria-selective cytotoxicity. However, their synergistic combination enables functional mitochondrial targeting through a binary mechanism involving (i) preferential enrichment of PpIX within tumor mitochondria following 5-ALA administration and (ii) irradiation-triggered induction and propagation of mitochondrial oxidative stress. We therefore propose this “binary approach” as a conceptual framework in which two individually non-mitochondria-targeting modalities converge to confer mitochondria-centered cytotoxicity. Although this framework was supported by experimental observations, it should be regarded as a hypothesis-driven model requiring further mechanistic validation. Nevertheless, it provides a biologically plausible rationale for understanding the selective vulnerability of tumor mitochondria to 5-ALA-RDT and for guiding future translational studies.

## 6. Initial ROS Production in 5-ALA-RDT

Although delayed ROS production contributes substantially to 5-ALA-RDT efficacy, the physicochemical mechanisms underlying the initial generation of ROS at the time of irradiation remain unclear. The ionizing radiation used in radiotherapy, including X-rays and γ-rays, interacts with matter via the photoelectric effect, Compton scattering, and pair production to generate secondary electrons [30]. These secondary electrons are central mediators of radiation-induced chemical reactions, particularly water radiolysis, which leads to the formation of highly reactive species such as hydroxyl radicals [44]. Porphyrinoid compounds, including PpIX, exhibit high electronic flexibility and redox activity [81]. PpIX enhances the initial generation of ROS, including hydroxyl radicals, superoxide anions, and singlet oxygen, via radiation-induced water radiolysis [33]. In this context, PpIX is not considered a passive bystander, but rather an active modulator of early redox reactions induced by ionizing irradiation.

A recent review has proposed that PpIX can accept radiolytically generated secondary electrons to form a radical anion (PpIX•^−^), which subsequently transfers electrons to nearby molecular oxygen to enhance superoxide production [30]. Importantly, subsequent protonation and competitive reactions with hydroxyl radicals may allow PpIX•^−^ to revert to its ground state, suggesting a redox-recycling process rather than irreversible molecular degradation. The proposed recycling mechanism is conceptually similar to the photochemical cycling of photosensitizers used in PDT, which supports sustained singlet oxygen generation under light exposure [37]. Cherenkov radiation generated during ionizing irradiation may, in principle, induce the photochemical excitation of PpIX [29]; however, current evidence indicates that its quantitative contribution is likely limited under clinically relevant radiotherapy conditions and is therefore unlikely to represent the dominant mechanism of ROS generation in 5-ALA-RDT [30].

Taken together, these observations suggest that during the initial ROS generation in 5-ALA-RDT, PpIX may act as a recyclable redox mediator that channels radiation-generated secondary electrons into a sustained mitochondrial ROS cascade. This cascade involves sequential superoxide formation, hydrogen peroxide generation, and Fe^2+^-dependent hydroxyl radical amplification via Fenton chemistry to increase oxidative stress without depleting PpIX [82]. Because 5-ALA-induced PpIX preferentially accumulates within the mitochondria, these early redox reactions are likely to be spatially confined to the mitochondrial compartment. Such spatial confinement fundamentally distinguishes 5-ALA-RDT from conventional radiotherapy, in which primary ROS are diffusely generated throughout the cells. Accordingly, we propose that the mitochondrial localization of the initial redox reactions represents a key mechanistic feature of 5-ALA-RDT, linking selective PpIX accumulation to sustained oxidative stress and downstream radiodynamic efficacy.

## 7. Target Cells for 5-ALA-RDT in Malignant Gliomas

In the preceding sections, we examined the interaction between tumor cells and ionizing irradiation, focusing on cell state-dependent vulnerability, nuclear DNA damage, and mitochondrial oxidative stress in the context of 5-ALA-RDT. While these experimental observations provide important mechanistic insights at the single-cell level, translating them directly into clinical decision-making remains challenging. In clinical practice, crucial information needed to define optimal treatment conditions and select patients for 5-ALA-RDT in malignant gliomas is largely absent. Definitive evidence regarding which tumor cell populations are most susceptible, their spatial distribution, and their relationship to conventional imaging or surgical findings has yet to be established. Accordingly, in this section, we adopt a hypothesis-driven interpretative approach that integrates accumulated clinical experience from 5-ALA-FGS, established knowledge from clinical neuro-oncology, and current understanding of 5-ALA metabolism and porphyrin biology. By synthesizing these independent but complementary lines of evidence, we aim to refine the conceptual understanding of potential target cell populations for 5-ALA-RDT and to provide a biologically grounded framework for its future clinical application, rather than definitive mechanistic conclusions.

A pivotal randomized, multicenter phase III trial of 5-ALA-FGS established the clinical value of 5-ALA as an intraoperative visualization tool for malignant glioma resection [13]. This landmark study demonstrated a significant improvement in the EOR and progression-free survival at 6 months in patients undergoing 5-ALA-FGS compared to those undergoing conventional white-light microsurgery. However, this increased EOR did not translate into a statistically significant prolongation of overall survival, underscoring the intrinsic limitations of surgery alone for the management of infiltrative gliomas. Subsequent systematic histopathological analyses of newly diagnosed GBMs provided critical insights into the biological meaning and limitations of 5-ALA-induced fluorescence [83]. Detailed pathological examinations revealed that tumor cells, including infiltrative glioma cells and areas of microvascular proliferation, were frequently present not only in strongly fluorescent regions but also in areas classified intraoperatively as weakly fluorescent (vague) or even non-fluorescent. These findings clearly demonstrated that the absence of visible fluorescence does not correspond to the absence of tumor tissue. Moreover, intraoperative assessment of 5-ALA fluorescence is inherently subjective and depends on macroscopic visual perception under an operating microscope. In addition, anatomical and functional constraints on the brain impose unavoidable limits on the EOR. To preserve neurological function, surgical resection must often be restricted to eloquent or deep-seated regions, even when the residual tumor tissue remains fluorescent. Consequently, complete surgical eradication of malignant glioma is not feasible without unacceptable neurological morbidity, irrespective of fluorescence guidance [84,85]. These clinical realities emphasize that the infiltrative tumor cells responsible for recurrence frequently remain beyond the reach of surgical resection. It is also important to recognize that 5-ALA-induced fluorescence is not strictly tumor-specific. Multiple studies have demonstrated that non-neoplastic brain lesions can exhibit visible PpIX fluorescence after 5-ALA administration [86,87,88]. Fluorescence has been reported in infectious lesions, inflammatory processes, demyelinating diseases, and other nonneoplastic, contrast-enhancing tissues [86,87,88]. Case-based analyses and literature reviews indicate that inflammatory cell infiltration, blood–brain barrier disruption, and altered heme metabolism contribute to false-positive fluorescence signals. These factors must be carefully considered when interpreting fluorescence findings and extending 5-ALA-based approaches beyond intraoperative visualization. From a biological perspective, infiltrating tumor cells that escape visual detection may still accumulate 5-ALA-derived PpIX at subvisual levels. This suggests that such cells remain metabolically targetable, even when they are surgically invisible. These observations highlight a fundamental limitation of fluorescence-guided resection and provide a strong rationale for developing therapeutic strategies capable of targeting residual and infiltrative tumor cell populations beyond the confines of surgical visualization.

Considering the clinical translation of 5-ALA-RDT, it is the safety profile of 5-ALA that is critical. Perioperative hypotension has been reported in the urological field as a characteristic adverse event associated with oral 5-ALA-based photodynamic diagnosis (PDD) during transurethral resection of bladder tumors. Comprehensive analyses have reported hypotension rates of approximately 8.1% under general anesthesia and up to 27.8% under spinal anesthesia, indicating a significant influence of anesthetic modality [89]. Advanced age and concomitant use of renin-angiotensin system inhibitors have been identified as risk factors in this setting [90]. Experimental studies suggest that PpIX generated from exogenously administered 5-ALA can induce vascular smooth muscle relaxation through the activation of soluble guanylate cyclase, leading to increased cyclic guanosine monophosphate signaling and vasodilation [90,91]. In contrast, extensive safety evaluations in the neurosurgical field have demonstrated a favorable tolerability profile for 5-ALA. Clinical studies involving patients with World Health Organization grade III/IV gliomas have shown that the oral administration of 5-ALA at standard (20 mg/kg) or higher doses does not result in a dose-dependent increase in adverse events [92]. Although transient hypotension has been observed in a minority of patients, its occurrence is not correlated with the 5-ALA dosage. Furthermore, a phase II study evaluating high-dose 5-ALA (≥40 mg/kg) in patients undergoing resection of newly diagnosed or recurrent GBM reported no clinically significant hypotension and no treatment-related adverse events according to Common Terminology Criteria for Adverse Events grade 2 or higher [93]. Collectively, these clinical data indicate that while 5-ALA-induced hypotension warrants awareness, the overall safety of 5-ALA is well established in neurosurgical oncology. Importantly, the combination of a favorable safety profile and robust tumor-selective accumulation in malignant gliomas provides a critical foundation for extending 5-ALA beyond intraoperative visualization for multimodal therapeutic applications, including 5-ALA-RDT, aimed at residual and infiltrative tumor cell populations.

In clinical neuro-oncology, magnetic resonance imaging (MRI) with gadolinium-based contrast agents is the gold standard for tumor diagnosis, treatment planning, and response assessment. In gliomas, contrast enhancement indicates the leakage of gadolinium through a disrupted blood–brain barrier (BBB) within the tumor tissue, which has traditionally been interpreted as a surrogate marker of malignancy [94]. More recently, this disrupted barrier was conceptualized as the blood–tumor barrier (BTB), leading to the development of contrast agents and imaging strategies that specifically target BTB permeability [95,96]. Although the BTB generally exhibits higher permeability than the intact BBB, its permeability is spatially heterogeneous and varies substantially across and within individual gliomas [95]. Recent large-scale clinical analyses highlighted the heterogeneity of gadolinium enhancement in GBMs. Contrast enhancement is observed in approximately 92% of all GBMs; enhancement rates differ markedly depending on the diagnostic criteria: 95.3% in histologically defined GBMs based on the WHO 2016 classification, but only 78.8% in molecularly defined IDH-wildtype GBMs according to the WHO 2021 classification [97]. These findings indicate that a substantial subset of molecular GBMs lacks overt gadolinium enhancement on MRI. Accordingly, contrast enhancement primarily reflects BTB permeability rather than tumor cell density or intrinsic malignant potential. This discordance underscores a fundamental limitation of MRI-based assessments and suggests that reliance on gadolinium enhancement alone may underestimate infiltrative or molecularly aggressive tumor components. With respect to the relationship between 5-ALA and the BBB, experimental studies have demonstrated that an intact BBB is largely impermeable to 5-ALA [98,99]. Although supraphysiological plasma concentrations of 5-ALA can result in limited passive diffusion across the intact BBB, its permeability remains extremely low [100,101]. Autoradiographic studies using radiolabeled 5-ALA have shown that, even under steady plasma concentrations, brain uptake reaches only approximately 1% of plasma levels within one hour after administration [101]. These data indicate that BBB disruption is a critical prerequisite for clinically relevant 5-ALA delivery to brain tissue. Supporting this concept, clinical studies have demonstrated that tumor detection with 5-ALA-induced PpIX fluorescence exhibits higher sensitivity and specificity than gadolinium-enhanced intraoperative MRI in the tumor border zone of malignant gliomas [102]. Thus, similar to gadolinium contrast agents, passage through a disrupted BBB or BTB is an essential first step in 5-ALA-derived fluorescence. However, the subsequent intracellular accumulation of PpIX reflects tumor-specific metabolic activity rather than vascular permeability alone. In a clinical study of WHO 2016 grade 2 gliomas, visible 5-ALA fluorescence was observed in 21.6% of cases [103]. Fluorescence positivity was associated with increased 18F-FET PET uptake and gadolinium enhancement but showed no correlation with molecular markers, including IDH mutation status, EGFR expression, p53 mutation, ATRX loss, or MGMT promoter methylation. Notably, overall survival was associated with age, IDH wild-type status, and 5-ALA fluorescence, whereas gadolinium enhancement itself showed no significant prognostic association [103]. Collectively, these observations suggest that gadolinium enhancement and 5-ALA fluorescence capture overlapping but biologically distinct tumor features— and vascular permeability versus heme-pathway-linked metabolic activity— and may therefore diverge, particularly in infiltrative or low-tumor-burden compartments. This discordance provides a strong mechanistic and clinical rationale for exploring 5-ALA-RDT as a complementary therapeutic strategy targeting residual and infiltrative tumor cell populations that are not reliably delineated by conventional imaging alone.

Mechanistically, the preferential accumulation of 5-ALA-induced PpIX in tumor cells is attributed to multiple, non-mutually exclusive processes, including (i) altered expression or activity of enzymes involved in the heme biosynthetic pathway [104,105], (ii) dysregulated membrane transporters mediating cellular 5-ALA uptake and PpIX efflux [106,107,108,109,110,111,112,113,114,115,116,117], (iii) aberrant intracellular iron metabolism affecting ferrochelatase activity [118,119,120,121], and (iv) impaired metabolic flux or turnover within the heme biosynthesis cascade [122,123]. Because PpIX is a downstream metabolite generated from exogenously administered 5-ALA, its intracellular accumulation is inherently dependent on the cellular metabolic context. Accordingly, PpIX levels are expected to vary substantially among GBM cells as a function of the intrinsic metabolic state, temporal dynamics following 5-ALA administration, and spatial localization within the tumor microenvironment, including the tumor core, invasive rim, and infiltrative margins. Importantly, these determinants are not static. Enzymatic activity, transporter expression, and iron handling can differ not only between patients but also among subpopulations of tumor cells within the same lesion, and may change dynamically in response to microenvironmental factors such as hypoxia, nutrient availability, and therapeutic stress. Thus, assigning causal primacy to a single mechanism governs PpIX accumulation is problematic. Instead, the observed fluorescence heterogeneity in malignant gliomas likely reflects the convergence of multiple metabolic programs that coexist, vary spatially, and evolve temporally within the tumor ecosystem.

While this section highlights the clinical and metabolic heterogeneity of 5-ALA fluorescence, a critical unresolved question remains: Which tumor cell populations are the most relevant targets for 5-ALA-based RDT? In malignant gliomas, most of the tumor burden responsible for recurrence consists of infiltrative tumor cells that escape both surgical resection and conventional imaging-based delineation. Glioma cells infiltrate the brain via characteristic patterns, including perivascular accumulation, perineuronal satellitosis, and subpial spread [3], and can extend far beyond MRI-defined tumor boundaries [124]. Consequently, postoperative radiotherapy remains the cornerstone for controlling residual disease in malignant gliomas [125].

Accumulating evidence indicates that GBM cells derived from the same tumor can adopt distinct molecular programs and biological behaviors, such as proliferation and migration, particularly at the interface between the tumor core and surrounding normal brain tissue, a phenomenon conceptualized by the “go-or-grow” theory [126]. Under this paradigm, effective therapy must address deeply infiltrative glioma cells within the normal brain tissue that differ molecularly and phenotypically from the resectable tumor bulk. Recent single-cell studies using human GBM specimens have elucidated the molecular differences between the tumor bulk (core and rim) and surrounding invasive margin [127,128].

A key methodological strength of these studies is the use of 5-ALA as a functional metabolic marker, enabling the identification of tumor cells based on 5-ALA metabolism rather than anatomical location or static molecular features. Within this framework, glioma cells exhibiting the intracellular accumulation of 5-ALA-derived PpIX are operationally defined as 5-ALA-positive cells. Notably, 5-ALA-positive cells at the invasive margin exhibit distinctive biological characteristics compared with those in the tumor bulk, including: (i) acquisition of a mesenchymal (MES) subtype associated with enhanced invasive capacity; (ii) activation of inflammatory wound-response programs linked to MES-like functional states and transcriptomic similarity to recurrent GBM; (iii) metabolic reprogramming with a shift toward glycolytic pathways to adapt to hypoxic stress; and (iv) close spatial and functional coexistence with myeloid cells, suggesting tumor-myeloid interactions that support MES phenotype acquisition and immune-modulated survival [127,128]. Importantly, although these marginal tumor cells are not necessarily highly proliferative, they appear to be biologically primed for recurrence by engaging mesenchymal, inflammatory, metabolic, and immune-interactive programs long before the recurrent lesions become detectable on MRI.

Collectively, these observations suggested that GBM cells residing in the infiltrative margin—, the population most responsible for recurrence—, can exhibit cellular states that enable 5-ALA metabolism and intramitochondrial PpIX accumulation. Because ionizing irradiation has superior tissue penetration compared to the optical irradiation used in PDT, 5-ALA-RDT provides a rational strategy for functionally targeting surgically unresectable and spatially dispersed infiltrative cells. This infiltrative compartment remains largely undetectable using conventional radiographic modalities, including MRI, which are inherently limited in their ability to identify early, low-burden infiltrative diseases. Accordingly, a conceptual shift from image-defined tumor volumes to functionally defined cellular states may be required to guide therapeutic targeting in GBM, analogous to non-genotoxic physical treatment modalities such as tumor-treating fields (TTFields) [129]. The binary approach proposed in this review is relevant to this framework. Neither 5-ALA-induced mitochondrial accumulation of PpIX nor ionizing irradiation alone constitutes a mitochondria-targeted therapeutic strategy. However, their combination in 5-ALA-RDT enables functional mitochondrial targeting through two complementary processes: (i) preferential enrichment of PpIX within tumor cell mitochondria driven by tumor-specific metabolic states, and (ii) spatially confined mitochondrial oxidative stress triggered by ionizing irradiation. Malignant gliomas are characterized by profound cellular, metabolic, and spatial heterogeneity, particularly within infiltrative tumor compartments that escape surgical resection and remain invisible to conventional imaging modalities. As discussed, infiltrative glioma cells—, often biologically primed for recurrence through mesenchymal, inflammatory, metabolic, and immune-interactive programs—, may retain the capacity for 5-ALA metabolism and mitochondrial PpIX accumulation despite being radiographically occult. From this perspective, 5-ALA functions not only as a surgical visualization aid but also as a functional cell-state marker that identifies tumor cell populations defined by metabolic vulnerability rather than anatomical boundaries. Although many aspects of 5-ALA metabolism, mitochondrial targeting, and radiodynamic susceptibility in gliomas remain unclear, the present synthesis suggests that this binary strategy provides a conceptual framework for treating infiltrative diseases beyond the limitations of image-guided approaches. By integrating metabolic cell-state information with the deep tissue penetration of ionizing irradiation, 5-ALA-RDT offers a biologically informed avenue for targeting residual and invasive glioma cells that are otherwise inaccessible to conventional therapies.

## 8. Concept for the Development of Radiosensitizers for RDT

As discussed above, the development of radiosensitizers for RDT has increasingly emphasized cellular and subcellular damage rather than direct nuclear DNA damage, reflecting a revised framework in contemporary radiation biology. Experimental studies have demonstrated the efficacy of multiple RDT-oriented radiosensitizers that operate through non-DNA-centered mechanisms [130,131,132]. Notably, most platforms are nanometal-based constructs rationally designed to integrate two core functions: (i) converting ionizing irradiation into ROS, either via secondary electron-mediated chemistry or scintillation-based energy conversion, and (ii) controlling subcellular localization, enabling the confinement of oxidative stress to specific compartments such as the mitochondria or the plasma membrane.

In the Hf-DBB-Ru system [DBB-Ru = bis(2,2′-bipyridine)(5,5′-di(4-benzoato)-2,2′-bipyridine)ruthenium(II) chloride], hafnium (Hf) nodes generate ROS via high atomic number (high-Z) effects, enhancing secondary electron production and hydroxyl radical generation, whereas the Ru-containing ligand confers mitochondrial affinity and promotes mitochondrial localization [130]. Thus, radiophysical ROS amplification and biological mitochondrial targeting can be combined on a single platform. Similarly, the CsI(Na)@MgO plus 5-ALA platform combines radiodynamic and photodynamic effects, in which CsI is both a high-Z ROS generator (predominantly hydroxyl radicals) and a scintillator emitting luminescence (~410 nm) under X-ray irradiation, which can excite 5-ALA-induced PpIX to generate singlet oxygen within tumor cell mitochondria [132]. Here, 5-ALA is not merely an adjunct photosensitizer but also acts as a functional cell-state marker and mitochondrial targeting element, exemplifying a prototypical binary approach.

Similarly, Rose Bengal (RB)-arginylglycylaspartic acid (RGD)-modified gold nanoclusters (AuNCs) function as high-Z radiosensitizers and scintillators, emitting luminescence at approximately 550 nm. The RGD moiety confers tumor selectivity by targeting integrins expressed on the plasma membrane of glioma cells. This architecture enables spatially confined oxidative stress in the plasma membrane while simultaneously facilitating singlet oxygen generation through RB-mediated photochemical reactions [131]. Recently, semiconductor-like charge trapping strategies have been introduced to generate an afterglow, prolong ROS generation after irradiation cessation, and extend the temporal window of radiodynamic effects [133]. In this context, physical dose amplification and biological targeting are decoupled yet functionally integrated.

Collectively, these examples illustrate that contemporary RDT radiosensitizer development is converging on a binary— or multicomponent design philosophy, in which radiophysical ROS generation is synergistically coupled with biological targeting at the cellular or subcellular level. This emerging conceptual framework has driven the rapid expansion of radiosensitizer RDT research and provides a foundation for mechanism-driven, biologically informed design strategies that extend beyond conventional DNA-centered radiosensitization.

## 9. Bench to Bedside Translation of 5-ALA-RDT

Despite the growing experimental interest in 5-ALA-RDT, both the mechanistic basis of 5-ALA-mediated radiosensitization and robust clinical efficacy data remain limited. Consequently, translating 5-ALA-RDT from the bench to the bedside presents substantial challenges. Nevertheless, an important distinction between 5-ALA and many experimental radiosensitizers is that its clinical safety has already been firmly established through its extensive use in FGS and PDD. Moreover, accumulating evidence suggests that 5-ALA functions not only as a photosensitizer but also as a functional cell-state marker that reflects tumor-specific metabolic and mitochondrial characteristics. From this perspective, 5-ALA-RDT holds promise as a cell-state-guided therapeutic strategy that may complement existing multimodal treatments for malignant gliomas. Rather than replacing conventional radiotherapy, 5-ALA-RDT may expand its biological scope by enabling the selective targeting of metabolically vulnerable tumor cell populations that are not adequately addressed by DNA-centered paradigms alone. To define clinically meaningful indications, irradiation timing, and treatment protocols for 5-ALA-RDT, a revised conceptual framework of radiotherapy that integrates advances in radiation delivery technology with emerging insights into mitochondrial biology and tumor cell-state heterogeneity is required.

5-ALA has been widely used in clinical practice, and its safety profile for FGS in malignant gliomas is well established. Radiotherapy has a long clinical history and is supported by extensive evidence in different tumor types. Technological advances have enabled the development of highly conformal radiotherapy modalities, including stereotactic radiotherapy (SRT), stereotactic radiosurgery (SRS), and intensity-modulated radiotherapy (IMRT), which can precisely control the dose distribution to minimize the exposure of healthy tissues and reduce toxicity. In particular, SRT and SRS deliver high radiation doses to small, well-defined intracranial targets with submillimeter accuracy, making them attractive platforms for biologically selective strategies such as 5-ALA-RDT [134,135,136].

Therefore, the integration of 5-ALA-RDT applied using the stereotactic techniques represents a clinically feasible and conceptually coherent strategy for the treatment of malignant gliomas. In supporting of this concept, a recent prospective study evaluated the efficacy of intraoperative balloon electronic brachytherapy (IBEB) in patients with recurrent GBM [137]. In that study, tumors were resected under 5-ALA fluorescence guidance, followed by the placement of a balloon applicator within the resection cavity. The tumor bed was then irradiated with a single 20-Gy dose using kilovoltage X-rays (~50 keV) delivered by a low-linear energy transfer (LET) system. The study reported a significantly longer median local progression-free survival in the IBEB group than in controls without IBEB [137]. Although this study did not directly evaluate the 5-ALA-mediated radiodynamic effects, the authors speculated that radiosensitization by endogenous porphyrins, including PpIX, might have contributed to the observed benefits [137].

From a radiation physics perspective, photon interactions in the kilovoltage range are dominated by the photoelectric effect, resulting in a localized, dense generation of low-energy secondary electrons [138]. In contrast, megavoltage X-rays predominantly interact via Compton scattering, producing higher-energy secondary electrons that travel longer distances and deposit energy more diffusely [138]. These differences yield distinct spatial patterns of energy deposition at the subcellular scale. In the context of 5-ALA-RDT, dense secondary electron generation with kilovoltage irradiation may facilitate efficient oxidative stress amplification in infiltrative glioma cells that accumulate 5-ALA-induced PpIX at the tumor-brain interface. Particle therapies (e.g., protons and carbon ions) are increasingly used clinically across tumor types and offer distinct radiobiological and physical dose-distribution advantages [139]. However, direct evidence for 5-ALA-mediated radiodynamic effects under particle irradiation remains sparse. One in vitro study has reported that 5-ALA and its downstream metabolite PpIX can enhance radiation responses under carbon-ion beam irradiation, accompanied by increased ROS generation and augmented γ-H2AX signals in tumor cells [28]. Notably, the subcellular distribution of porphyrins may influence dominant biological targets, potentially modulating the balance between nuclear DNA damage and organelle-centered oxidative stress. These observations suggest the feasibility of extending radiodynamic concepts beyond photon radiotherapy, while underscoring the need for systematic validation under clinically relevant particle-beam conditions.

Although fractionated irradiation may be advantageous for accumulating radiodynamic effects and driving ROS beyond cytotoxic thresholds in 5-ALA-RDT, clinical observations from IBEB suggest that single-dose irradiation could also achieve meaningful effects under appropriate physical and biological conditions. In PDT, it is well established that even when the total delivered light energy is equivalent, irradiation at a low fluence rate can result in more efficient singlet oxygen generation and enhanced cytotoxicity—a phenomenon referred to as metronomic PDT [37,140]. By analogy, in 5-ALA-RDT, equivalent absorbed radiation doses (Gy) may not necessarily yield equivalent biological effects, because photons generated at different energies engage in distinct physical interaction processes. These differences affect the production, density, and spatial distribution of secondary electrons, which are critical determinants of intracellular ROS generation. Therefore, optimization of 5-ALA-RDT protocols should consider not only the total dose and fractionation schedules but also the radiation modality and photon energy, as these parameters govern secondary electron density and the spatial confinement of oxidative stress. Mechanistic and translational studies that explicitly integrate these physical variables into mitochondrial biology and tumor cell- state dynamics are essential for rational clinical protocol development.

## 10. Conclusions and Future Perspectives

Interactions between porphyrinoid compounds, including 5-ALA-induced PpIX, and ionizing radiation have been recognized for decades. However, these interactions have only recently been re-examined within a coherent biological framework and conceptualized as RDT.

5-ALA-RDT is an emerging therapeutic concept that challenges the conventional DNA-centered paradigms of radiotherapy by introducing mitochondria-centered oxidative vulnerability and functional cell-state targeting. Although experimental and clinical evidence remains limited, the collective data reviewed herein support the biological plausibility of this approach, particularly for malignant gliomas characterized by profound spatial, metabolic, and cellular heterogeneity.

A central implication of this review is that the principal therapeutic targets of 5-ALA-RDT are not necessarily the radiographically defined tumor bulk, but rather infiltrative glioma cell populations that escape surgical resection and remain undetectable by conventional MRI. These cells, often residing beyond gadolinium-enhancing regions—, may nonetheless retain active 5-ALA metabolism and intramitochondrial PpIX accumulation, rendering them susceptible to radiodynamically amplified oxidative stress. From this perspective, 5-ALA-RDT offers a strategy for functionally targeting residual diseases that lie outside the scope of anatomy-based treatment planning. In contrast to contemporary high-precision radiotherapy, which relies on the accurate delineation of MRI-defined target volumes, the biologically relevant “target field” in 5-ALA-RDT is determined by the spatial distribution of 5-ALA-induced PpIX. Consequently, this approach dose not require high-precision radiation delivery. Instead, simplified irradiation systems tailored for 5-ALA-RDT—that are potentially compact, cost-effective, and adaptable—may represent a feasible strategy for improving accessibility while preserving biological selectivity. Importantly, 5-ALA-RDT is not intended to replace the established standards of care for malignant gliomas, including maximal resection and concomitant radiochemotherapy. Given that 5-ALA is a naturally occurring heme precursor with a favorable safety profile, the key challenge is to determine how 5-ALA-RDT can be optimally integrated into current and emerging multimodal treatment strategies. Based on currently available evidence, several clinically relevant scenarios merit consideration. First, the observation that fractionated irradiation can amplify the radiodynamic effects suggests that 5-ALA-RDT could be explored as an adjunct to standard postoperative radiochemotherapy for newly diagnosed GBMs which confirmed by histological evaluation, following maximal safe resection. In this setting, repeated administration of 5-ALA in combination with fractionated irradiation may sustain mitochondrial oxidative stress in infiltrative tumor cells, thereby enhancing therapeutic vulnerability and potentially contributing to an extension of progression-free and overall survival in GBMs. Second, intraoperative or perioperative irradiation strategies—, such as cavity- or brachytherapy-based irradiation—, may offer additional opportunities to exploit radiodynamic effects under conditions of high local dose delivery and favorable physical interactions. Importantly, these potential clinical applications remain hypothesis-driven and require systematic experimental and clinical validation. It must be emphasized that, at present, neither the optimal radiation modality, dose, fractionation schedule, nor patient selection criteria for 5-ALA-RDT have been established. Moreover, direct causal evidence linking delayed mitochondrial ROS amplification to tumor-specific lethality remains limited. These limitations underscore the need for a revised experimental and conceptual framework that explicitly incorporates mitochondrial dynamics, redox kinetics, and functional tumor heterogeneity into radiobiological research. Nevertheless, in contrast to many experimental radiosensitizers, 5-ALA possesses a unique translational advantage: its clinical safety has already been extensively validated, and its tumor-selective metabolic accumulation is well characterized. These attributes make 5-ALA-RDT a realistic candidate for early-phase clinical exploration, once biologically informed irradiation protocols have been defined. Progress in this field will therefore depend not only on accumulating additional experimental data but also on reframing the conceptualization of radiotherapy to target tumor biology beyond nuclear DNA damage alone.

In conclusion, although 5-ALA-RDT cannot yet be regarded as an established therapeutic modality, it provides compelling proof-of-concept for a mitochondria-inclusive, cell-state-guided approach to radiotherapy. Advancements in this strategy require the integration of radiation physics, mitochondrial biology, and clinical neuro-oncology within a revised radiobiological framework. A prospective phase I/II clinical trial investigating 5-ALA-RDT for recurrent GBM was recently initiated, representing an important translational milestone [141]. Beyond malignant gliomas, this emerging strategy may ultimately prove applicable to multiple tumor types, underscoring the need for continued mechanistic, technological, and clinical investigations.

## Data Availability

The data analyzed in the current review are accessible from the corresponding references.

## Abbreviations

ATP	adenosine triphosphate
AuNCs	modified gold nanoclusters
CDK2	cyclin-dependent kinase 2
DAMPs	damage-associated molecular patterns
Drp1	dynamin-related protein 1
EOR	extent of resection
FGS	fluorescence-guided surgery
GBM	glioblastoma
IBEB	intraoperative balloon electronic brachytherapy
ICD	immunogenic cell death
IMRT	intensity-modulated radiotherapy
MC	mitochondrial catastrophe
MRI	magnetic resonance imaging
mtDNA	mitochondrial DNA
PDT	photodynamic therapy
Plk1	Polo-like kinase 1
PpIX	protoporphyrin IX
PpIX-TLST	PpIX triplet-state lifetime technique
RB	Rose Bengal
RDT	radiodynamic therapy
RGD	arginylglycylaspartic acid
ROS	reactive oxygen species
SRS	stereotactic radiosurgery
SRT	stereotactic radiotherapy
5-ALA	5-aminolevulinic acid
5-ALA-RDT	5-ALA-based radiodynamic therapy
